# Ovarian cyst impacted in the pouch of Douglas at 20 weeks' gestation managed by laparoscopic ovarian cystectomy: a case report

**DOI:** 10.1186/1752-1947-3-7257

**Published:** 2009-05-12

**Authors:** Fawzia Sanaullah, Ashwini K Trehan

**Affiliations:** 1 Dewsbury and District Hospital, Halifax Road, Dewsbury WF13 4HS, UK

## Abstract

**Introduction:**

The frequency of ovarian cysts in pregnancy is reported to be 1 in 1000 pregnancies. Laparoscopic ovarian cystectomy has been described in the literature as case series but this is the first case where an ovarian cyst at 20 weeks' gestation impacted in the pouch of Douglas was managed exclusively by laparoscopy.

**Case presentation:**

A 31-year-old primigravida woman was diagnosed as having an 11 cm ovarian cyst at 20 weeks' gestation. At the 19th week routine ultrasound scan, the mass appeared to be cystic with no solid component. She was asymptomatic. In view of the size of the cyst, options were discussed with her including laparoscopic ovarian cystectomy which she agreed to. Laparoscopic surgery during pregnancy is reported to be safe and beneficial for pregnant women. At laparoscopy, a transvaginal scan was performed to localize the cyst and an ovarian cystectomy was carried out. The patient had an uneventful recovery and subsequent antenatal period. She had a lower segment caesarean section for non-progress of labour when both ovaries were found to be normal and mobile.

**Conclusions:**

Laparoscopic surgery during pregnancy has numerous advantages compared to open laparotomy. This is a rare example of an ovarian cyst in the pouch of Douglas impacted behind the uterus which was managed by laparoscopy and shows the safety of the technique in the presence of an expert laparoscopic surgeon.

## Introduction

The frequency of ovarian cysts in pregnancy is reported to be 1 in 1000 pregnancies. The authors present a patient with an ovarian cyst impacted in the pouch of Douglas at 20 weeks' gestation. It was managed by laparoscopic ovarian cystectomy.

## Case presentation

A 31-year-old Caucasian woman was booked into our hospital for her first pregnancy. An ultrasound scan at 19 weeks confirmed a normal fetal anatomy and a large simple septate cyst arising from the pelvis measuring 11 cm. Neither of the ovaries was seen, so it was difficult to determine the origin of the cyst. The cyst was not complex and was reported to be a simple cyst with a single thin septum. Although both ovaries were not visualized, which is not unusual at 19 weeks, because of the relationship of the cyst with the uterus and the cyst being in the pouch of Douglas, it was suggested to be of probable ovarian origin. The overall morphological features of the mass did not indicate malignancy. In view of the large size and septation of the cyst, the surgical option to remove the cyst by the laparoscopic technique was discussed with the patient, which she agreed to.

She was admitted at 20 weeks' gestation. She was asymptomatic and the height of the fundus at 20 weeks corresponded to 26 weeks' gestation. After induction of general endotracheal anaesthesia, a nasogastric tube was passed to remove any gaseous distension of the stomach. The uterine fundus was palpated and a Verres needle was inserted through the Palmer's point. After insufflation with CO_2_ to a pressure of 20 mmHg, a 5 mm cannula was placed through the same site. A 10 mm infra-umbilical port was inserted. Two secondary ports, 5 mm each, were inserted under direct vision, the right and left lateral ports at the level of the umbilicus. Normally, right and left ports are inserted but it was impossible to reach the left side from the right port, therefore a further left port was inserted between the left lateral port and the Palmer's point port. The two left port placements facilitated exposure of the ovarian cyst, adhesiolysis of the bowel from the left adnexa, avoided potential injury to the gravid uterus and minimized uterine manipulation.

At laparoscopy, the gravid uterus was seen with normal right ovary with no ascites, smooth peritoneal surface, normal upper abdomen and no cyst could be seen. The left adnexa was obscured by congenital adhesions of the sigmoid colon and omentum. These are normally congenital adhesions from the sigmoid colon to the sidewall around the pelvic brim. Transvaginal ultrasound was therefore performed at this stage. The ultrasound scan confirmed an 11 cm simple cyst with thin septum in the pouch of Douglas behind the uterus. The bowel adhesions to the left adnexa were divided. The cyst was still not visible so the operating table was tilted towards the right which deflected the uterine fundus away from the midline. At this point, a small part of the left ovarian cyst was visualized between the pelvic side wall and the gravid uterus. The cyst was aspirated with a Verres needle avoiding intraperitoneal spill and 300 ml of serous fluid was drained initially. Once the cyst had shrunk, the ovary was pulled out from the pouch of Douglas. The remaining cyst was then completely aspirated (nearly 500 ml of more fluid). The pouch of Douglas was visualized, and looked normal. The ovarian cystectomy was performed by dissecting away the cyst wall from the ovarian tissue by stripping and sharp scissors dissection. The remaining ovarian tissue was refashioned with 3/0 PDS by purse string suture burying the ovarian edges. The pouch of Douglas and peritoneal cavity were washed with Ringer's lactate. The intra-abdominal pressure was maintained below 12 mmHg throughout the procedure. Theatre occupancy time for the whole procedure was 120 minutes. No tocolytics were used as there is no evidence for any role of tocolytics at this gestational age. Prophylactic antibiotics were administered. The fetal heart was auscultated before and after the procedure.

Her postoperative recovery was uncomplicated and she was discharged home the following day. She was readmitted 12 days later due to anxiety and constipation resulting in some spasmodic abdominal cramps. She settled with reassurance, simple analgesia and laxatives. The patient was reviewed in the clinic 2 weeks later and discharged to routine antenatal care. Her subsequent antenatal course was uncomplicated. Histology of the cyst wall confirmed a mucinous cyst adenoma. She was admitted at 39 weeks in spontaneous labour and due to non-progress of labour, she had an emergency lower segment caesarean section. At operation, both her ovaries were normal and mobile. The baby was male and weighed 3.7 kg, with Apgar score 7 at 1 minute and 9 at 5 minutes.

## Discussion

Laparoscopic cystectomy in pregnancy was first reported in 1991 by Nezhat *et al.*[1] and then a second case in 1994 by Howard and Vill [2]. Since then, for various reasons, laparoscopic surgery in pregnancy has rapidly increased as surgeons realized the safety of the technique in general as well as in pregnancy. Pregnancy is no longer considered as an absolute contraindication for laparoscopic procedures. Currently, there are almost 150 case reports of laparoscopic surgery in pregnancy in the literature. However, we believe that this is the first reported case at 20 weeks' gestation with ovarian cyst impacted in the pouch of Douglas where intraoperative transvaginal scanning, lateral tilt of the operating table and bowel adhesiolysis facilitated ovarian cystectomy.

The frequency of ovarian tumours is about 1 in 1000 pregnancies [3] and those which are malignant represent about 1 in 15,000 to 32,000 pregnancies [4]. Corpus luteum cyst and benign cystic teratoma contribute two-thirds of the cases. A typical corpus luteum cyst is <3 cm in diameter and usually resolves. Ovarian cysts with diameter ≥6 cm which persist or enlarge beyond 16 weeks' gestation, are at risk of complications and need tissue diagnosis and, therefore, surgical evaluation [5]. Most surgical options for adnexal masses in pregnancy are managed ideally in the second trimester after organogenesis is complete decreasing the risk of fetal loss, eliminating the 15% to 20% background risk of spontaneous miscarriage and allowing for spontaneous regression of the mass.

Management of the adnexal mass, whether it be conservative or surgery, remains controversial. Surgical removal is considered to reduce the risk of undiagnosed malignancy, torsion, infection, rupture, haemorrhage and obstruction of labour. These complications may necessitate emergency surgery which carries a higher risk of fetal wastage compared with elective surgery. Furthermore, the risk of obstruction of labour is calculated to be 17% to 21% [6]. Operative procedures ranging from aspiration of the cyst to oopherectomy are described in the literature. In this patient, the cyst was impacted in the pouch of Douglas and the risk of obstruction of labour was avoided by antenatal laparoscopic ovarian cystectomy.

Once the decision is made for surgical management, the specific approach is the next consideration. Until recently, most of these procedures were performed by exploratory laparotomy. There is evidence to suggest that laparoscopy and laparotomy do not differ with regard to fetal outcome, that is, fetal weight, gestational age, growth restriction, infant survival and fetal malformations [7]. However, the major advantages of laparoscopy are magnification and panoramic view of the pelvis resulting in reduced intra-operative uterine manipulation which may lead to decreased postoperative uterine irritability, miscarriage rate and preterm labour which is seen in 50% of third trimester cases with an open approach. There are several reports on the safety of the laparoscopic procedure for gynaecologic and non-gynaecologic surgery such as cholecystectomy and appendicectomy during the second trimester of pregnancy with no increase in miscarriage rate. The reduced postoperative pain, rapid recovery as well as the other described typical advantages after laparoscopic surgery are of potential benefit to pregnant women and may encourage more widespread use of this procedure in pregnant women [8]. In addition, the cosmetic results are much better and the discomfort of stretching and distension of the laparotomy scar due to the rapidly growing uterus is avoided.

The possible risks of laparoscopy include compression of uterine blood flow through elevated intra-abdominal pressure, fetal acidosis, fetal exposure to carbon monoxide from coagulation and uterine injury from cannula placement. The risks of hypercarbia and acidosis are reduced by keeping the operating time short and pressures as low as possible - no higher than 15 mmHg. Ventilation of the lungs needs to be carefully monitored and constantly adjusted to compensate for the pneumoperitoneum and positional changes. For operative laparoscopy, adequate exposure is possible with low pressure, although a short period of higher pressure increases the safety of port insertion [9,10].

Although laparoscopic surgery during pregnancy is becoming more common, it is rarely performed late in the second trimester. The enlarged uterus makes surgery more challenging. Laparoscopic cystectomy up to 27 weeks' gestation has been reported when the cyst contents were aspirated and the cystectomy was performed after exteriorization [11]. Moreover, very few case series provide long-term follow-up. Only one series with 11 cases of 1 to 8 years of follow-up has reported no evidence of developmental or physical abnormalities in the resultant children [12].

The laparoscopic entry technique is less of a concern in the first trimester because the pregnant uterus remains in the pelvic cavity. However, with increasing gestational age, the uterus rises out of the pelvis and there is an increasing chance of injury while inserting the Verres needle. Generally, open cannulation laparoscopy or Palmer's point entry is recommended for laparoscopy during pregnancy. This avoids the risk of penetrating injury to the pregnant uterus by either the Verres needle or the trocar cannula [13].

In the case series and reports in the literature, there is usually no difficulty in exposing the adnexal mass as the enlarged pregnant uterus tends to displace it towards the top of the uterus. Mathevet *et al.* described a series 48 laparoscopic procedures performed in 47 patients with adnexal masses in pregnancy and two cases required laparotomy due to dense adhesions and difficulty with haemostasis [14]. However, in our patient, the cyst was hidden, impacted behind the uterus and not easily visible, and an extra port on the left side facilitated adhesiolysis of the bowel without manipulating the uterus. Tilting the operating table and aspiration of the cyst fluid before full retrieval of the cyst avoided laparotomy.

Large cystic masses may require decompression to fit though a small incision. By decompressing a cyst into a laparoscopic bag, spillage can be minimal or non-existent. Copious irrigation also helps to keep the residual content to a minimum [15]. After cystectomy, the ovarian incision can be left open or approximated by three techniques: fine monofilament suture of the edges, tissue glue or coagulation of the ovarian cortex adjacent to the surface, which will in some instances evert the edges. We closed the ovarian incision with 3/0 prolene because the cyst wall was large and left a large ovarian incision. Moreover, stitching was necessary to avoid adhesions between the raw ovarian surface and the raw peritoneal surface left after bowel adhesiolysis in the left adnexa [15].

## Conclusions

This case demonstrates that, at 20 weeks' gestation, an ovarian cystectomy is possible for an 11 cm cyst impacted in the pouch of Douglas using the laparoscopic approach and with a surgeon skilled in advanced laparoscopic techniques.

## Consent

Written informed consent was obtained from the patient for publication of this case report. A copy of the written consent is available for review by the Editor-in-Chief of this journal.

## Competing interests

The authors declare that they have no competing interests.

## Authors' contributions

The case was managed and operated by AKT. The literature search and case writing were carried out by FS. The authors have read and approved the final manuscript.
